# Arthroscopic debridement improves range of motion for heterotopic ossification after total knee replacement: a retrospective cohort study

**DOI:** 10.1038/s41598-024-56300-1

**Published:** 2024-03-11

**Authors:** Dong-Liang Zhang, Wei Zhang, Yi-Ming Ren, Wen-Jun Zhao, He-Jun Sun, Zheng-Wei Tian, Meng-Qiang Tian

**Affiliations:** 1 Department of Joint and Sport Medicine, Tianjin Union Medical Center, Tianjin Medical University, Nankai University Affiliated People’s Hospital, Jieyuan Road 190, Hongqiao District, Tianjin, 300121 People’s Republic of China; 2 Nursing Department, Tianjin Union Medical Center, Nankai University Affiliated People’s Hospital, Tianjin, People’s Republic of China

**Keywords:** Total knee replacement, Heterotopic ossification, Arthroscopy, Range of motion, Revision, Acute inflammatory arthritis, Musculoskeletal abnormalities, Osteoarthritis

## Abstract

The presence of heterotopic ossification (HO) after primary total knee replacement (TKR) is rare and associated with limited mobility and stiffness of the knee. This study aimed to identify if the arthroscopic debridement after TKR could decrease HO and improve the function and range of motion. Thirty HO patients after TKR were retrospectively separated into 2 cohorts. 15 patients of group A accepted the arthroscopic debridement, while 15 patients of group B only had non-operative treatment, mainly including oral nonsteroidal anti-inflammatory drugs (NSAIDs) and rehabilitative treatment. Visual analog scale (VAS) scores, knee society knee scores (KSS), range of motion (knee flexion and knee extension) were obtained before treatment and at 1 month, 3 months, and 6 months after treatment. Radiography of after-treatment was also evaluated to assess the changes in HO. There were 3 males and 27 females with a mean age of 67.4 ± 0.8 years in group A and 68.2 ± 1.3 in group B. The onset time of HO was 3–6 months. The maximum size of the ossification was < 2 cm in 23 knees, 2 cm < heterotopic bone < 5 cm in 6 knees and > 5 cm in 1 knee. The size of HO decreased gradually in all knees by X-ray film at the last follow-up. There were no significant differences in VAS scores after replacement between two groups (*p* > 0.05). The average range of motion preoperatively in group A was − 15.2–90.6°, which postoperatively increased to − 4.2–110.0°. Meanwhile, the KSS scores and average range of motion of the group A were better than those of the group B at each follow-up time after treatment. Arthroscopic debridement can decrease HO seen from postoperative X-rays, improve the function and range of motion, as well as the pain remission between two groups are comparable. Consequently, arthroscopic resection of HO after TKR is recommended as soon as there is aggravating joint stiffness.

## Introduction

In recent years, roughly 15% of Americans have symptomatic knee arthritis, and in 2010 roughly 600,000 total knee replacements (TKRs) were performed^1^. The increasing number of artificial joint replacement makes people pay more and more attention to various complications after the operation. Heterotopic ossification (HO) is one of the common complications after artificial joint replacement. HO refers to the phenomenon of new bone formation in non-calcified tissues and mature lamellar bone in the soft tissues around joints under normal conditions^2,3^. HO after total hip arthroplasty is a common complication, approximately 43% and as high as 90% in high-risk patients, but new bone formation after primary TKR is a rare event^4^. An incidence of 1% to 42% has been reported in the literature^5^. The reason why HO is not emphasized is that most patients are asymptomatic. Only a small number of patients will experience pain, limited mobility and stiffness of the knee^6^.

At present, there is still a lack of sufficient understanding of the pathogenesis of HO. In 1965, Urist et al. found that decalcified bone matrix can induce the formation of HO, and proposed that bone morphogenetic induction protein is the real inducer^7^. Chalmers et al. proposed three conditions necessary for HO formation: osteogenic inducing agent, osteogenic precursor cells, and tissue environment allowing osteogenesis. Sametimely, they considered that the formation of HO depends on the balance of local and systemic factors that stimulate and inhibit osteogenesis. Gender, age, disease factors, intraoperative factors and prosthesis types maybe all risk factors for its occurrence^8^.

At present, multiple treatment modalities are available for the treatment of the HO around the knee, including non-operative and operative treatments. The non-operative methods to prevent and treat HO mainly include nonsteroidal anti-inflammatory drugs (NSAIDs), phosphate, manipulation under anesthesia, local radiation therapy, etc.^9,10^. However, clinically HO with obvious symptoms including patients with stiffness after TKR, often cannot improve symptoms by oral NSAIDs and other non-operative means^11^. With the prolongation of symptoms, patients often lose patience with conservative treatment and seek further surgical treatment. Operative treatments include arthroscopic debridement, revision TKR, and others^12,13^. However, surgical treatment of HO after TKR has rarely been reported. The purpose of this study was to evaluate the efficacy of arthroscopic debridement regarding the postoperative range of motion for HO after TKR. The hypothesis was that the arthroscopic debridement significantly improves range of motion for symptomatic HO after TKR.

## Materials and methods

### Ethics approval and consent to participate

The local Ethics Committee for Research on Human Beings of Tianjin Union Medical Center approved the study and original data collection. The ethical approval statement and the need for informed consent were waived by the local Ethics Committee for Research on Human Beings of Tianjin Union Medical Center for this manuscript because the individual data was fully anonymized. All methods were performed in accordance with the relevant guidelines and regulations of Helsinki declaration of 1964 and its amendments.

### Patient population

From January 2010 to December 2021, 30 HO patients underwent TKR with limited mobility of the knee or stiffness of the knee in our hospital were collected retrospectively. 15 of them accepted the arthroscopic debridement (group A), and 15 patients refused to undergo surgery and only had non-operative treatment, mainly including oral NSAIDs and rehabilitative treatment. The surgical indication is that all patients have obvious knee joint pain with limited mobility or stiff, and X-rays show obvious ectopic ossification. Limited mobility or stiff patients were defined by preoperative flexion less than 100° or greater than 10° flexion contracture. Range of motion was measured in a standardized fashion. Extension and flexion were measured using a goniometer at all visits and range of motion was calculated by the operating surgeon (DLZ), and additional measurements were performed by a fellowship-trained orthopedic surgeon (YMR) for interobserver consistency. Radiographs were independently reviewed by 2 joint surgery fellowship-trained orthopedic surgeons (MQT, ZWT), with all disagreements resolved by a third orthopedic surgeon (WJZ). Preoperative and postoperative radiographs were additionally compared, in conjunction with a review of the operative reports, to determine whether HO was excised.

The inclusion criteria were as follows: (1) All HO patients retrospectively collected in this study had symptoms and chief complaints of pain and limited mobility; (2) HO was identified as bone spurs from the anterior surface of the distal femur that were not seen pre-operatively, most commonly seen on lateral radiographs; (3) patients with rheumatoid arthritis, osteoarthritis or hypertrophic arthrosis. The exclusion criteria of the present study were defined as: (1) patients with diffuse idiopathic skeletal hyperostosis or ankylosing spondylitis; (2) patients had a history of hormone injection or infection; (3) preoperative stiff knees; (3) serious limited mobility and stiffness of the knee or postoperative stiffness due to other reasons.

### Surgical technique

Adopting the anterolateral approaches of the knee joint, the incision is about 0.5 cm long respectively, and blunt separation is used to release the front of the knee joint. By exploring the knee joint, it is found that the joint fluid in the joint cavity is clear, without purulent exudation, and a large number of bone hyperplasia and fibrous scar cover under the patella and in front of the tibia. These bone hyperplasia and fibrous scar were released and removed with a planer, and the knee joint prosthesis and gasket were exposed after proper biting. The prosthesis and gasket are not loose and in good position, Under the arthroscopy, it was found that synovitis and hyperplasia were filled between the femoral prosthesis and the spacer, and a large number of bone and soft tissues were proliferated around the tibial prosthesis, in the intercondylar fossa and under the patella. After full biting and loosening, the knee joint flexion and extension activities were no longer blocked, and the mobility was improved. After the skin was sutured, one branch of tranexamic acid was injected into the joint to inhibit bleeding, and the elastic bandage was wrapped. The operation was successful, and about 10 ml of blood was lost during the operation^14–17^.

### Clinical evaluation and follow-up

The patients completed a questionnaire consisting of a 10-point visual analog scale (VAS) (0–10, with 0 reflecting no pain) for knee pain preoperatively and at each follow-up visit. Functional outcomes were scored preoperatively and at each follow-up visit according to the knee society knee score (KSS), range of motion (knee flexion and knee extension). Additionally, radiography after treatment was also evaluated to assess the outcomes of the procedures. Office follow-ups were conducted before-treatment, after-treatment (the follow-up time that patient's first follow-up score after surgery or conservative treatment), 1 month, 3 months, 6 months after the treatment.

### Statistical analysis

The statistical analysis was performed with the use of SPSS version 22.0. The results of descriptive data analysis are shown as means ± standard deviations for continuous variables, and as frequencies and percentages for categorical variables. Student t-tests were used for analysis of changes in mean age, onset time of HO after TKR, follow-up, VAS score, KSS score, range of motion. Fisher’s exact test was used for sex, radiographic grading system of HO, prosthesis, notching, and effusion. All *p* values ≤ 0.05 were considered statistically significant.

## Results

### Baseline characteristics

**Figure 1:**
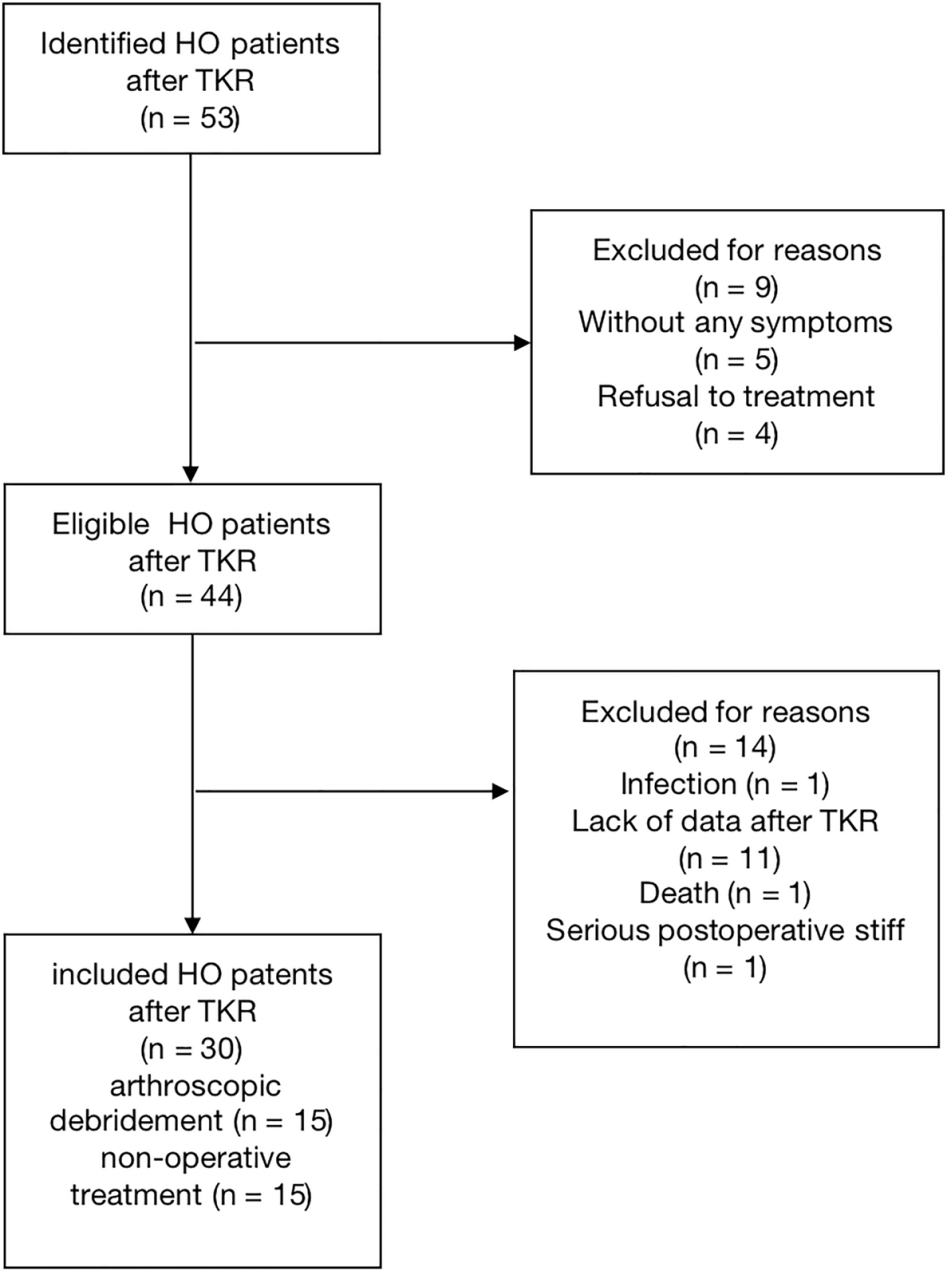
Flow chart of HO patient screening.

The demographic results of the patients are summarized in Table 1. 15 patients in group A and 15 patients in group B met the inclusion and exclusion criteria and flow chart was showed in Fig. 1. There were 3 males and 27 females with a mean age of 67.4 years in group A and 68.2 in group B. The average duration of follow-up time was 6.1 months in group A and 6.0 months in group B. The onset time of HO was 3–6 months after operation, and there was no significant increase 6 months after operation. The preoperative diagnoses were osteoarthrosis (29 knees) and rheumatoid arthritis (1 knee). 30 knees all implanted cemented prosthesis. Four patients were treated with posterior stabilized prosthesis (Zimmer biomet Inc, Vanguard, USA), one patient was treated with posterior stabilized prosthesis (Link Inc, Gemini MKII, Germany) and 25 patients were treated with posterior stabilized prosthesis (DePuy Orthopaedics Inc, PFC sigma, USA).

**Table 1:** Demographic data.

Characteristics	Group A	Group B	*p*-Value
Number of patients	15	15	
Mean age (year)*	67.4 ± 0.8	68.2 ± 1.3	0.052
Sex (male/female)^#^	2/13	1/14	> 0.999
Diagnosis^#^			> 0.999
Osteoarthrosis	14	15	
Rheumatoid arthritis	1	0	
Radiographic grading system of HO^#^			0.390
Type I	10	13	
Type II	4	2	
Type III	1	0	
Prosthesis^#^			> 0.999
Cementless	0	0	
Cemented	15	15	
Notching^#^	0	1	> 0.999
Effusion^#^	2	5	0.390
Onset time of HO after TKR (mo)*	4.6 ± 2.5	4.2 ± 1.9	0.626
Follow-up (mo)*	6.1 ± 2.2	6.0 ± 1.5	0.885

^#^ Fisher's exact test.

*Student t-test.

*HO* heterotopic ossification; *TKR* total knee replacement; *mo* month.

**Figure 2:**
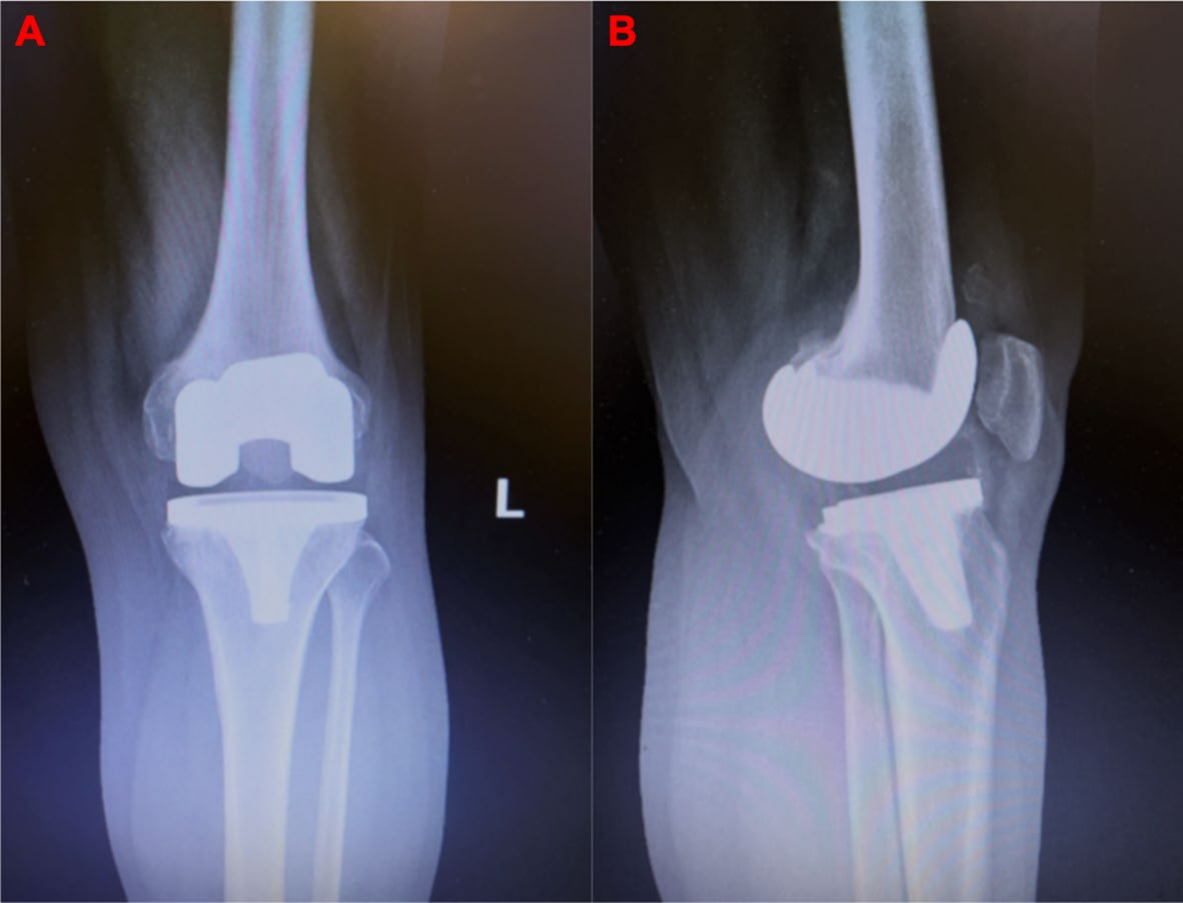
Postoperative TKR AP view (**A**) and lateral view (**B**) radiograph of patient showing type I HO (less than 2 cm of new bone formation).

**Figure 3:**
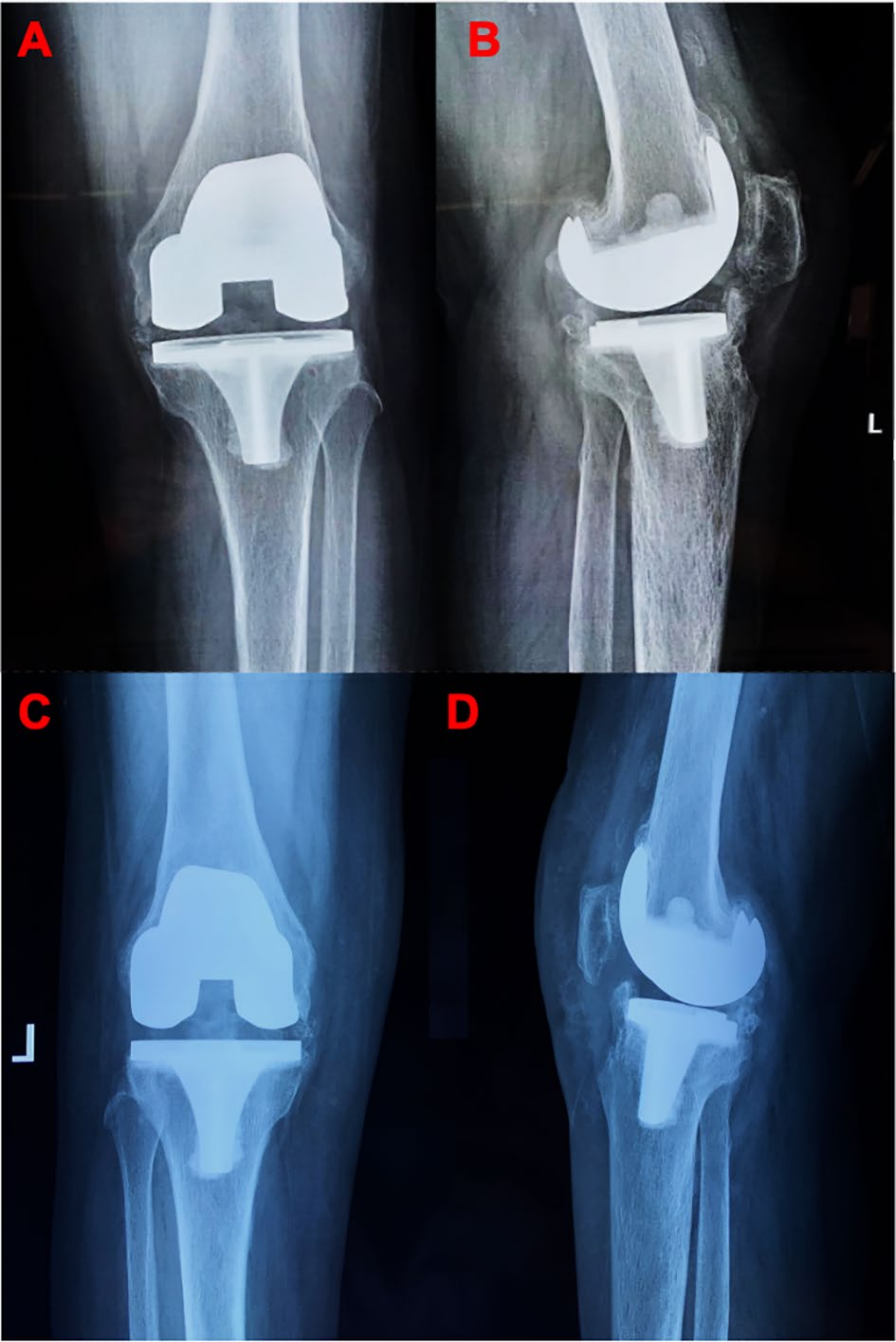
Postoperative TKR AP view (**A**) and lateral view (**B**) radiograph of the patient, showing type II HO (more than 2 cm of new bone formation). Postoperative TKR AP view (**C**) and lateral view (**D**) radiograph of patient accepted the arthroscopic debridement at the 3 months follow-up.

**Figure 4:**
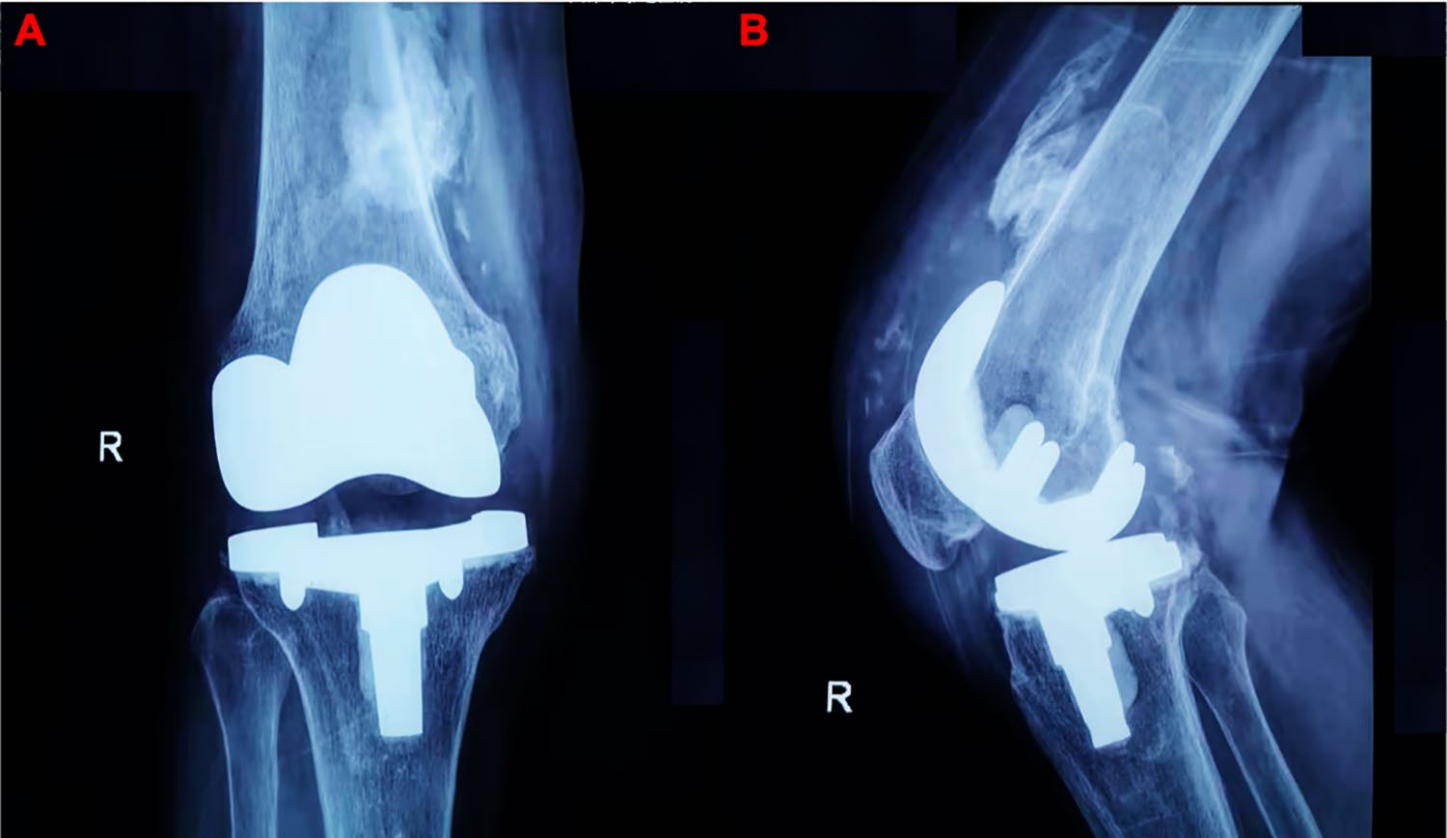
Postoperative TKR AP view (**A**) and lateral view (**B**) radiograph of patient, showing type III HO (more than 5 cm of new bone formation).

Three grades of HO were defined based on the size of the largest segment of bone^18^. 23 patients with heterotopic bone < 2 cm measured by X-ray film belong to grade I HO after TKR (grade I < 2 cm, 2 cm ≤ grade II < 5 cm, grade III ≥ 5 cm), 6 patients with 2 cm ≤ heterotopic bone < 5 cm measured by X-ray film belong to grade II HO after TKR, and 1 patient with heterotopic bone ≥ 5 cm measured by X-ray film belong to grade III HO after TKR. There were seven HO patients with joint effusion after operation, and the fluid was extracted with negative cultures. X-rays of typical patients are shown in Figs. 2, 3, 4.

### Clinical outcomes

**Figure 5:**
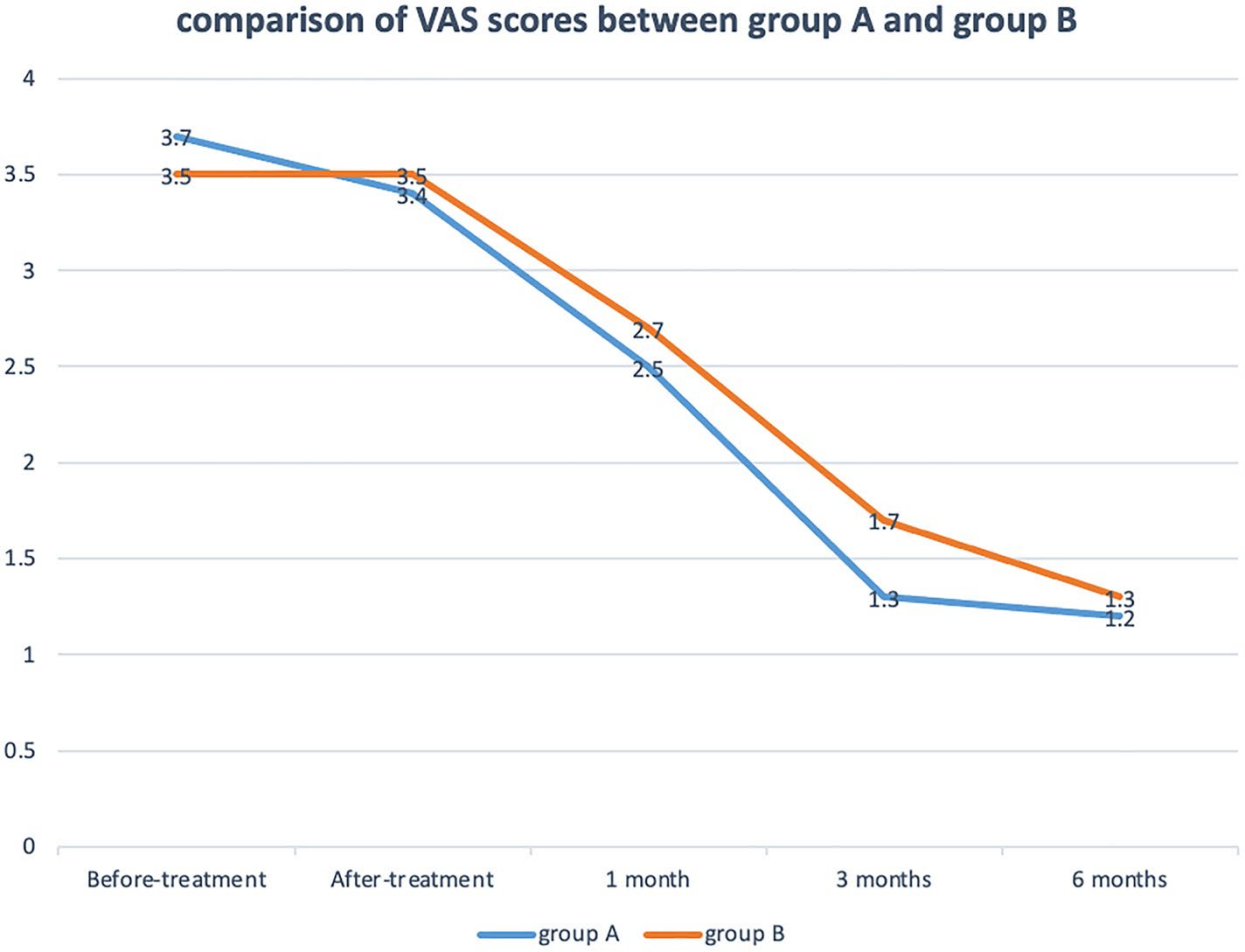
Visual analog scale (VAS) pain score for patients receiving between the arthroscopic debridement (group **A**) and non-operative treatment (group **B**). *p < 0.05 for the difference between the groups.

**Figure 6:**
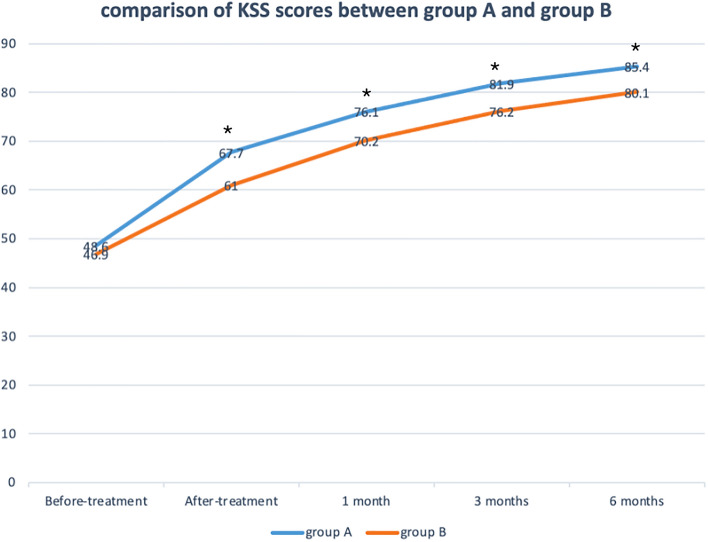
Knee society knee score (KSS) function score for patients receiving between the arthroscopic debridement (group **A**) and non-operative treatment (group **B**). *p < 0.05 for the difference between the groups.

There were significant decreases of VAS scores between before-treatment and different time points after-treatment in Fig. 5. No statistically significant differences in VAS scores at each follow-up time were observed between the groups. There were significant increases of KSS scores between before-treatment and different time points after treatment. KSS scores increased more in group A than group B with significance at follow-up of after-treatment (67.7 ± 7.4 vs 61.0 ± 6.7, *p* = 0.0147), 1 month (76.1 ± 7.0 vs 70.2 ± 7.8, *p* = 0.0378), 3 months (81.9 ± 6.8 vs 76.2 ± 6.4, *p* = 0.0252) and 6 months (85.4 ± 7.3 vs 80.1 ± 6.9, *p* = 0.0475) in Fig. 6.

**Figure 7:**
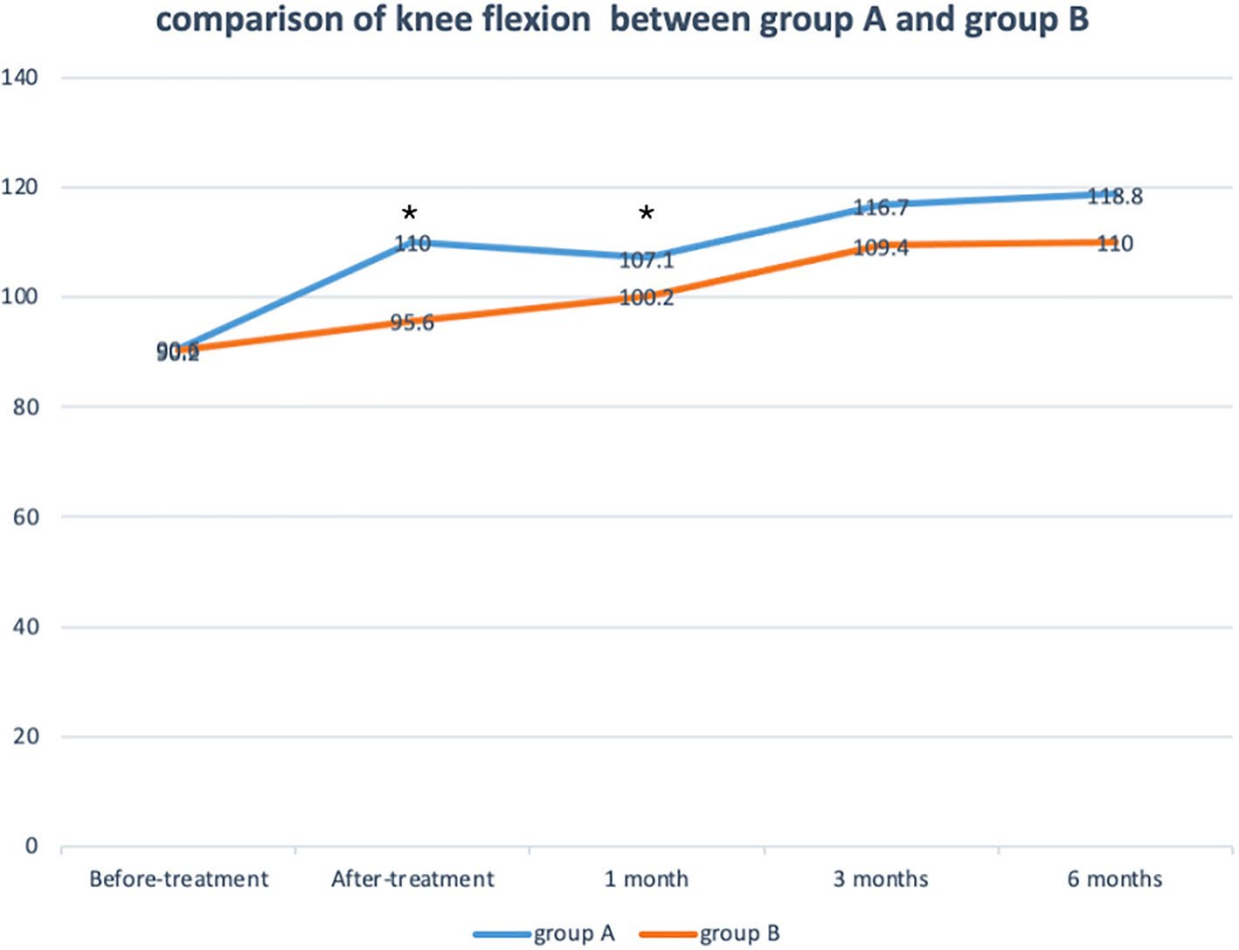
Comparison of knee flexion for patients receiving between the arthroscopic debridement (group **A**) and non-operative treatment (group **B**). *p < 0.05 for the difference between the groups.

**Figure 8:**
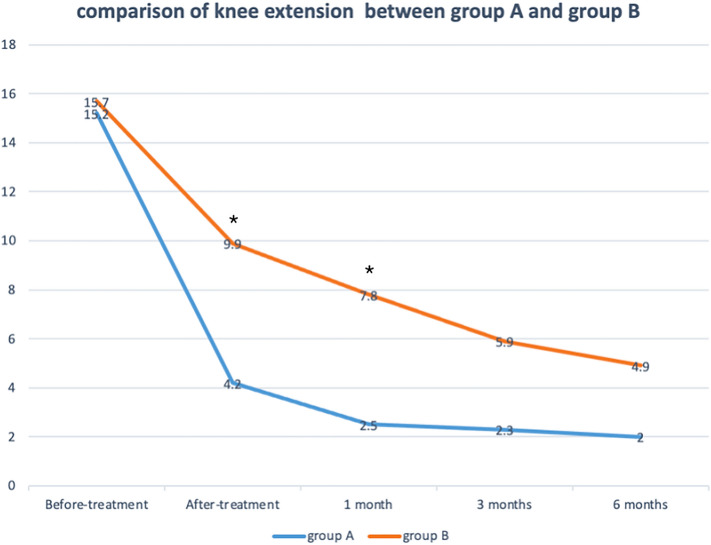
Comparison of knee extension for patients receiving between the arthroscopic debridement (group **A**) and non-operative treatment (group **B**). *p < 0.05 for the difference between the groups.

The mean flexion angle of knee before-treatment was 90.6 ± 13.1° in group A and 90.2 ± 14.3° in group B, and the extension angle of knee before-treatment was − 15.2 ± 12.4° in group A and − 15.7 ± 13.5° in group B. The flexion angle of knee after-treatment in group A had no significant difference compared with that of group B at 3 months and 6 months after treatment, but it was higher than that of group B at after-treatment follow-up (110.0 ± 14.7° vs 95.6 ± 12.9°, *p* = 0.0081) and at 1 month follow-up (107.1 ± 14.3° vs 100.2 ± 13.8°, *p* = 0.0283) in Fig. 7. Within group B, patients of group A had higher extension angle at after-treatment follow-up (-4.2 ± 7.1° vs − 9.9 ± 7.8°, *p* = 0.0455) and at 1 month follow-up (-2.5 ± 7.2° vs − 7.8 ± 6.9°, *p* = 0.0490), but at 3 months and 6 months after treatment they had no significant difference in Fig. 8. The original score data is shown in the Table S1.

### Radiography after treatment

The HO are mostly located in front of the distal femur, with the width of the bottom of the heterotopic bone base > 1 cm. It is fixed in the femoral cortex, showing a peak, ball or irregular shape, and invades the quadriceps femoris or extends to the distal femur. Excepting the above distribution, HO of two knees are located in front of the tibial plateau, near the distal end of the patellar tendon. One patient with notching after operation was reported by X-ray film. The size of HO decreased gradually in all knees at the last follow-up.

Serial radiography was available for 18 patients (13 in group A, 5 in group B) and showed minimal or scattered HO after treatment. In total, 12 slightly reduced HO (2 in group A, 10 in group B) were reported as dictated in the operative report or observed on radiographic comparison of before-treatment and after-treatment in Table 2. No negative HO was showed by serial radiographs at the last follow-up.

**Table 2:** Summary of postoperative radiography after excision of H0.

		Group A	Group B
Radiography of last follow-up			
	Negative HO	0	0
	No films	0	0
	Minimal HO	9	2
	Scattered HO	4	3
	Slightly reduced HO	2	10

*HO* heterotopic ossification.

## Discussion

Large heterotopic bone leads to joint pain and limited extension and flexion, which affects the clinical effect of joint replacement^19,20^. The findings of this retrospective cohort study indicate that arthroscopic debridement can reduce HO observed in postoperative X-rays, enhance the function and range of motion.

The formation of HO is closely related to its own osteogenic ability. Takashi et al. have proved that the incidence rate of postoperative HO is positively correlated with the size of hyperplastic osteophytes. He believes that the potential osteogenic ability can promote the generation of postoperative HO^5^. Bone morphogenetic protein (BMP) has a significant ability to induce osteogenesis. Animal experiments have proved that BMP transplanted outside the skeletal system can form ectopic bone. In humans, the overexpression of BMP-4 caused by the mutation of BMP-4 gene is an important reason for ectopic ossification^21^. The authors believe that removal of osteophytes during operation and excessive stripping of periosteum resulting in the release of osteoinductive factors and stem cells are also the reasons for accelerating the formation of HO. The X-ray films show that the heterotopic bone are mostly located in front of the distal femur. This part is the location of femoral osteotomy and the starting point of HO. The periosteum of this part is damaged during osteotomy, and then the osteogenic ability is significantly increased to promote the formation of heterotopic bone. In this study, 7 patients had joint effusion within 1 week after operation. There was no relevant report on the relationship between joint effusion and hematocele and the formation of HO. However, hematoma organization in soft tissue can also lead to HO. Postoperative infection is also an important reason for the formation of HO^22^. Barack et al. confirmed that the incidence of HO in infected joints could reach 34%^23^. In this study, the two main diagnoses related with HO treatment were osteoarthritis in 29 (96.7%) cases and rheumatoid arthritis in 1 case (3.3%). One patient with notching after operation was reported by X-ray film. Roth et al. revealed that patients with rheumatoid arthritis are at lower risk of HO than patients with osteoarthritis. An impairment of wound healing would appear to promote the development of a HO. Notching and hypertrophic arthrosis are highly likely to be associated with the development of a bony spur in the ventral contact area of the prosthesis^24^, and women with hypertrophic arthrosis are especially prone to HO postoperatively^25^. Revision TKR is particularly concerning for the generation of HO, given the additional soft tissue and bone trauma associated with these procedure^26^. Further researches of HO after TKR should focus on the formation mechanism of HO and effective preventive measures to further reduce the incidence rate of serious HO.

HO changes with time after TKR, forms in a short time and remains stable for a long time. HO entered the bone formation stage 2–3 weeks after operation, and developed on X-ray film 4–6 weeks after operation. Christof et al. found that the ectopic bone did not increase after 3 months postoperatively by following up 615 patients with TKR for 1–6 years^24^. The time of HO in this experiment was 3–6 months (average 4.6 ± 2.5 in group A and 4.2 ± 1.9 in group B) after operation, and there was no significant increase at 6 months after operation. Some scholars believe that HO after TKR is a self-limiting complication. For small ectopic ossification without clinical symptoms, it donot need be treated after eliminating the stimulating factors. In order to prevent the occurrence of severe HO, a variety of preventive measures can be taken to avoid excessive damage to soft tissue during operation. Perioperative anticoagulation therapy and postoperative closed drainage can help to reduce the occurrence of HO. NSAIDs are more definite preventive drugs. Christof et al. found that the incidence rate of HO was significantly reduced by taking any two kinds of NSAIDs, steroids and aspirin within 2 weeks after operation^24^. The main mechanism of NSAIDs in preventing HO is to inhibit cyclooxygenase, prevent prostaglandin synthesis, reduce local inflammatory response, and inhibit the transformation of mesenchymal cells into osteoblasts^27^.

All the 30 patients with HO had joint pain after operation, especially when flexing and going upstairs and downstairs. This was because the friction between heterotopic bone and soft tissue during joint flexion caused inflammatory stimulation, which would cause local swelling and joint effusion in severe cases. The symptoms of joint pain were not relieved when HO was formed and stabilized^28^. At each follow-up time after treatment in our study, the pain scores of arthroscopic debridement groups were lower than that of the control groups, which proved that HO was an important cause of joint pain. All patients with HO had limited range of motion, and the range of motion at after-treatment follow-up and at 1 month follow-up was significantly higher than that in the control groups. Meanwhile, our study showed KSS function scores of arthroscopic debridement groups increased at each follow-up time after treatment. There was significant limitation of knee extension function, mainly because the HO in the quadriceps femoris caused the weakening of quadriceps femoris muscle strength and inflammatory reaction. The limitation of flexion function is mainly due to the pain caused by ectopic bone friction, which stimulating soft tissue during knee flexion. In these cases, even passive rehabilitation exercise is not effective, and finally results in joint stiffness. For postoperative joint stiffness caused by quadriceps femoris adhesion, the range of motion can be restored by passive flexion exercise under anesthesia. However, joint stiffness caused by severe HO may lead to swelling of suprapatellar bursa, early stiffness of quadriceps femoris, and then inflammatory edema of soft tissue. The patient cannot extend or flex the joint. If passive rehabilitation under anesthesia is adopted, the inflammation of quadriceps femoris will be aggravated and the joint mobility will not be improved. Therefore, it is necessary to carry out surgical treatment for HO that causes clinical symptoms or limits extension and flexion^25,29^.

Surgical resection is the basic treatment. Cobb et al.^30^ reported significant range-of-motion increases after excision of HO. Chidel et al.^31^ reported a series of five patients (six knees) who underwent surgical excision of HO at 5 to 11 months after TKR, followed by single-dose radiation (700 Gy) prophylaxis on postoperatively first day. There was no recurrence of HO after radiation prophylaxis, and range of motion improved in all patients. Open surgical resection of ectopic ossification is more traumatic. Although the resection is more thorough, the soft tissue damage during the operation is large, which is easy to cause the recurrence of ectopic ossification. In this study, knee arthroscopic debridement was used to treat HO after TKR, with less trauma and high patient acceptance. Meanwhile, joint effusion and possible hematoma can be cleared simultaneously during arthroscopy. By retrospectively including 30 HO patients and comparing with the conservative treatment group, the knee arthroscopic debridement can effectively improve the range of motion and function of HO patients after TKR. The choice of operation time is the most critical. Premature resection of immature HO is easy to cause bleeding and recurrence. Ideal operation time included: no local fever or swelling; alkaline phosphatase (AKP) was normal (reflecting osteoblast activity); bone scan is normal or close to normal (reflecting bone metabolic activity)^32^. Some scholars considered that heterotopic bone formation should not be removed until at least 14 months after TKR. The connective tissue with ossification potential between heterotopic bone and surrounding muscle should be removed at the same time, otherwise it is easy to relapse^28,33^.

There are several limitations in this study. First, because this is a single-center retrospective study, and all HO patients were collected in a single research center. Due to the small sample size and limited follow-up time, the results may be biased. For example, HO is more common in men after hip replacement^22^, the high proportion of women (90%) in the present study concerning total knee replacement may act as a source of bias. On the one hand, it may be due to the selective bias caused by the retrospective collection of cases in this study, which resulted in gender differences. On the other hand, it is due to regional or ethnic differences. For example, the distal femur of Chinese women is narrower than that of white women, and some studies have compared the knee geometry of Asians and Caucasians, finding that their femur has different aspect ratios. However, most joint prosthesis designs are unable to perfectly cater to Asian women. Slight mismatches in prosthesis installation may lead to ectopic ossification^34–36^. Second, in view of the fact that only three types of knee cement fixed prosthesis are used in this study, it is still unclear whether the selection and fixation method of prosthesis in TKR has an impact on HO. In this retrospective study, The types of prosthesis and fixation methods were retrospectively obtained and not actively selected. The reason for the selection of prostheses was more often attributed to bias in the selection of a single prosthesis in a single research center. Currently, the vast majority of prosthesis fixation methods are bone cement fixation. The selection and fixation methods of prostheses as risk factors for HO are difficult to determine, which needs further study. Lastly, radiotherapy is also an important and effective method in the conservative treatment of HO and postoperative radiation could be used as prophylaxis against recurrence of HO. In view of the uncertain complications of radiotherapy and the cost of hospitalization, patients refused to receive radiotherapy. Therefore, none of the patients included in this study received radiotherapy.

## Conclusion

In summary, the results of this retrospective cohort study suggest that arthroscopic debridement can decrease HO seen from postoperative X-rays, improve the function and range of motion, as well as the pain remission between two groups are comparable. Consequently, arthroscopic resection of HO after TKR is recommended as soon as there is aggravating joint stiffness. Obviously, further study about preventing recurrence of HO on larger series and longer follow-up periods are mandatory.

### Supplementary Information

Supplementary Table S1.

## Data Availability

The patients’ data were collected in the Tian Union Medical Center. The datasets used and/or analysed during the current study available from the corresponding author on reasonable request.

## Abbreviations

HO: Heterotopic ossification
TKR: Total knee replacement
NSAIDs: Nonsteroidal anti-inflammatory drugs
VAS: Visual analog scale
KSS: Knee society knee score
AKP: Alkaline phosphatase
BMP: Bone morphogenetic protein
