# Supplementary material for: Arthroscopic debridement improves range of motion for heterotopic ossification after total knee replacement: a retrospective cohort study
Source: Sci Rep. 2024 Mar 11;14:5882. doi: 10.1038/s41598-024-56300-1 (PMC10928176; doi:10.1038/s41598-024-56300-1)
Supplement: Supplementary file 1 — Supplementary Table S1. [file 41598_2024_56300_MOESM1_ESM.doc]

|  |  |  |  |  |  |  |  |  |
| --- | --- | --- | --- | --- | --- | --- | --- | --- |
| **Table S1. Clinical outcomes according to the parameters** | | | | | | |  |  |
| Parameters | Follow-up |  | Group A(n=15) |  |  | Group B(n=15) |  | P-Value |
| VAS | Before-treatment |  | 3.7±1.3 |  |  | 3.5±1.0 |  | 0.6404 |
|  | After-treatment |  | 3.4±1.0 |  |  | 3.5±1.5 |  | 0.8315 |
|  | 1 month |  | 2.5±1.9 |  |  | 2.7±1.2 |  | 0.7329 |
|  | 3 months |  | 1.3±0.7 |  |  | 1.7±0.8 |  | 0.1561 |
|  | 6 months |  | 1.2±0.5 |  |  | 1.3±0.4 |  | 0.5501 |
| KSS | Before-treatment |  | 48.6±8.2 |  |  | 46.9±7.9 |  | 0.5677 |
|  | After-treatment |  | 67.7±7.4 |  |  | 61.0±6.7 |  | 0.0147 |
|  | 1 month |  | 76.1±7.0 |  |  | 70.2±7.8 |  | 0.0378 |
|  | 3 months |  | 81.9±6.8 |  |  | 76.2±6.4 |  | 0.0252 |
|  | 6 months |  | 85.4±7.3 |  |  | 80.1±6.9 |  | 0.0475 |
| Knee flexion | Before-treatment |  | 90.6±13.1 |  |  | 90.2±14.3 |  | 0.9369 |
|  | After-treatment |  | 110.0±14.7 |  |  | 95.6±12.9 |  | 0.0081 |
|  | 1 month |  | 107.1±14.3 |  |  | 100.2±13.8 |  | 0.0283 |
|  | 3 months |  | 116.7±12.9 |  |  | 109.4±12.5 |  | 0.1267 |
|  | 6 months |  | 118.8±12.3 |  |  | 110.0±13.4 |  | 0.0714 |
| Knee extension | Before-treatment |  | -15.2±12.4 |  |  | -15.7±13.5 |  | 0.9166 |
|  | After-treatment |  | -4.2±7.1 |  |  | -9.9±7.8 |  | 0.0455 |
|  | 1 month |  | -2.5±7.2 |  |  | -7.8±6.9 |  | 0.0490 |
|  | 3 months |  | -2.3±6.7 |  |  | -5.9±8.1 |  | 0.1954 |
|  | 6 months |  | -2.0±7.7 |  |  | -4.9±7.2 |  | 0.2958 |
| VAS=indicates visual analog scale, KSS=Knee Society knee score. | | | | | | | | |
| *Statistically significant differences between the groups by student t-test. | | | | | | | |  |
